# Development of a new ultra sensitive real-time PCR assay (ultra sensitive RTQ-PCR) for the quantification of HBV-DNA

**DOI:** 10.1186/1743-422X-7-57

**Published:** 2010-03-12

**Authors:** Dimitrios Paraskevis, Apostolos Beloukas, Catherine Haida, Antigoni Katsoulidou, Zisis Moschidis, Helen Hatzitheodorou, Agoritsa Varaklioti, Vana Sypsa, Angelos Hatzakis

**Affiliations:** 1 Department of Hygiene Epidemiology and Medical Statistics, Medical School, University of Athens, Athens, Greece; 2 Laikon General Hospital, 2nd Blood Transfusion Center, Athens, Greece

## Abstract

**Background:**

Improved sensitivity of HBV-DNA tests is of critical importance for the management of HBV infection. Our aim was to develop and assess a new ultra sensitive in-house real-time PCR assay for HBV-DNA quantification (ultra sensitive RTQ-PCR).

**Results:**

Previously used HBV-DNA standards were calibrated against the WHO 1^st ^International Standard for HBV-DNA (OptiQuant^® ^HBV-DNA Quantification Panel, Accrometrix Europe B.V.). The 95% and 50% HBV-DNA detection end-point of the assay were 22.2 and 8.4 IU/mL. According to the calibration results, 1 IU/mL equals 2.8 copies/mL. Importantly the clinical performance of the ultra sensitive real-time PCR was tested similar (67%) to the Procleix Ultrio discriminatory HBV test (dHBV) (70%) in low-titer samples from patients with occult Hepatitis B. Finally, in the comparison of ultra sensitive RTQ-PCR with the commercially available COBAS TaqMan HBV Test, the in-house assay identified 94.7% of the 94 specimens as positive versus 90.4% identified by TaqMan, while the quantitative results that were positive by both assay were strongly correlated (*r *= 0.979).

**Conclusions:**

We report a new ultra sensitive real time PCR molecular beacon based assay with remarkable analytical and clinical sensitivity, calibrated against the WHO 1^st ^International standard.

## Background

Chronic hepatitis B virus (HBV) infection can be assessed by evaluating clinical features and biochemical, virologic, and histologic parameters. Knowledge of HBV-DNA viral load is useful for predicting disease prognosis, determining infectivity, evaluating indications for treatment, assessing response to treatment identifying emergence of resistance, and diagnosing occult HBV infection [1-5].

There are several commercially available HBV-DNA tests. The oldest versions suffered from poor sensitivity (approximately 5·10^5 ^copies/mL) and a narrow dynamic range of HBV-DNA quantification (~3-4 log_10_), while the current limit of detection of the newer assays is lower than 50 IU/mL with a dynamic range of approximately 8 log_10 _[6-13]. Additionally several in-house quantitative assays for HBV-DNA have been developed, based mostly on real-time PCR methodology, showing a remarkable sensitivity and a wide linear range for quantification [6,7,11-14].

Importantly, improved sensitivity of HBV-DNA tests is of critical importance for the management of HBV infection as well as for the diagnosis of occult HBV infection [HBsAg(-), HBV-DNA(+)] [15].

The objective of this study was to develop a new ultra sensitive real-time PCR assay based on the knowhow of the existing real-time PCR assay for the quantification of the HBV-DNA [12] and, also, to assess its performance characteristics.

## Methods

### DNA extraction

The HBV-DNA was extracted from 0.5 mL of serum/plasma using the QIAamp UltraSens Virus Kit (QIAGEN Inc., Valencia CA) and the DNA was eluted in 60 μl of elution buffer. For each ultra sensitive RTQ-PCR HBV-DNA quantification measurement, a positive HBV-DNA sample with known HBV-DNA titer (~10^4 ^IU/mL) and one negative control (negative human plasma) (Procleix^® ^Negative Calibrator, Chiron/GenProbe, CA) were extracted additional to the unknown samples.

### Ultra Sensitive RTQ-PCR

The assay was carried out into a LightCycler^® ^2.0 apparatus. The reaction mixture contained 1× reaction buffer (LightCycler DNA Master HybProbe; Roche Applied Science. Germany), 5 mM MgCl_2_, 0.5 μM primer hbv305 (sense) 5'-GCCAAAATTCGCAGTCCC-3', 0.5 μM primer hbv460 (antisense) 5'-GATAGTCCAGAAGAACCAACAAGAAG-3', 0.4 μM molecular beacon 5'-CGCGCGATGAGGCATAGCAGCAGGATGAAGAACGCGCG-3' labelled with FAM and Dabcyl at the 5' and 3' ends, respectively, Taq DNA polymerase, dNTP mix (with dUTP instead of dTTP), and 1 U of uracil-DNA glycosylase (Roche Applied Science. Germany). The amplification profile was as follows: One cycle of initial denaturation (95°C for 10 min) followed by 40 cycles of amplification at 95°C for 0 sec, 55°C for 10 sec, and at 72°C for 8 sec. The final reaction volume was 60 μl containing 30 μl of extracted viral DNA.

### Analytical Sensitivity of the ultra sensitive RTQ-PCR

The sensitivity together with the 95% and 50% HBV-DNA detection limits of the assay were estimated by using the WHO 1^st ^International Standard for HBV-DNA (OptiQuant^® ^HBV-DNA Quantification Panel, Accrometrix Europe B.V.), tested in 8 replicas. We performed serial dilutions with human negative plasma (Procleix^® ^Negative Calibrator, Chiron/GenProbe, CA) of the WHO standard at final concentrations of 100, 50, 25, 10 and 5 IU/mL. The 95% and 50% HBV-DNA detection limits were calculated by probit analysis (8 replicas).

### Assessment in a low titter HBV-DNA panel

The clinical performance of the assay was tested on 27 samples drawn from 22 individuals with occult Hepatitis B (HBs Ag negative, consistently reactive by multiplex Procleix Ultrio HIV-1/HCV/HBV and Procleix Ultrio discriminatory HIV and HCV negative) screened with the discriminatory HBV assay (dHBV) (Chiron/GenProbe, Emeryville/San Diego, CA).

### Calibration of the HBV-DNA values copies/ml vs IU/ml

The calibration of the HBV-DNA values was performed against the WHO 1^st ^International Standard for HBV-DNA (OptiQuant^® ^HBV-DNA Quantification Panel, Accrometrix Europe B.V.). Specifically, serial dilutions of the WHO standard ranging from 2·10^2 ^to 2·10^6 ^IU/mL) in 5 individual sample preparations were tested by the ultra sensitive RTQ-PCR method. Based on a linear regression, a conversion formula was calculated for the in-house measurements (copies/mL) to the international standard units (IU/mL).

### Comparison of the ultra sensitive RTQ-PCR vs the previous real-time PCR assay

To compare the ultra sensitive RTQ-PCR assay with the previously reported test, HBV-DNA derived from 25 randomly selected samples was quantified using both methods. The correlation of the HBV-DNA quantifications by using the current and the previous in-house assay was also estimated.

### Comparison of the ultra sensitive RTQ-PCR vs COBAS TaqMan HBV Test

To compare the ultra sensitive RTQ-PCR assay with the commercially available COBAS TaqMan HBV Test, plasma samples collected from 94 different HBV-infected patients were tested. The correlation of the HBV-DNA quantifications by using the in-house and the commercial test was also estimated.

### Statistical analysis

Pearson's correlation coefficient was used to assess the strength of the linear association between the log_10_-transformed values of the estimated (copies/mL) versus the expected values (IU/mL) and also between HBV-DNA quantifications using the two in house real-time PCR assays and COBAS TaqMan as well. To compare the quantitative results obtained for the samples found positive by the two different methods (ultra sensitive RTQ-PCR vs COBAS TaqMan HBV Test) the fitted regression line was compared to the line of equality by testing the two-tailed hypothesis of slope = 1 and the intercept = 0.

## Results

The ultra sensitive RTQ-PCR was performed in a LightCycler^® ^2.0 apparatus using a plasmid HBV-DNA standard as described previously. We should note that the specificity of the assay was 100% as shown previously [12]. The differences between the newly developed method (ultra sensitive RTQ-PCR) and the previous one [12] were: 1) the utilization of a molecular beacon instead of 2 hybridization probes, 2) extraction of HBV-DNA from a larger volume of serum/plasma (0.5 versus 0.2 mL), 3) use of 30 μl instead of 10 μl extracted DNA, 4) modified RTQ-PCR conditions, and 5) all experiments were performed in a LightCycler^® ^2.0 apparatus because the latter shows improved performance characteristics compared to LightCycler^® ^1.0. Moreover reaction volume in LightCycler^® ^2.0 can be as large as 100 μL compared to 20 μL in the LighCycler^® ^1.0.

### Analytical sensitivity of ultra sensitive RTQ-PCR

To determine the analytical sensitivity of the ultra sensitive RTQ-PCR, 5 dilutions of the WHO 1^st ^International Standard for HBV-DNA were tested (OptiQuant^® ^HBV-DNA Quantification Panel, Accrometrix Europe B.V.). DNA concentrations above 25 IU/mL were detected on all 8 occasions; concentrations of 10 IU/mL were tested on 4 of 8 occasions (50%), while 5 IU/mL were detected on 2 out of 8 occasions (25%). The 95% and 50% detection end-point of the assay were 22.2 and 8.4 IU/mL respectively.

### Performance of ultra sensitivity RTQ-PCR assay in low titer HBV-DNA clinical samples

The performance of the assay was tested on 27 samples collected from 22 individuals with occult Hepatitis B (HBsAg negative, HBV-DNA consistently reactive by Ultrio, Ultrio HIV and HCV negative). This analysis was part of another study concerning the molecular typing of occult Hepatitis B in blood donors across Greece [15]. The characteristics of these patients are shown in Table 1. These samples were selected because of the low HBV-DNA titer. Among the 27 samples tested with the dHBV test, 19 (70%) were found to be above the threshold of detection, while a similar sensitivity was observed for the ultra sensitive RTQ-PCR method (18 samples tested positive out of 27, 67%). The mean HBV-DNA was 59.1 IU/mL (range, 10.2 to 346.7 IU/mL). We should note, however, that the dHBV assay was applied multiple times to 18 samples (a sample was considered positive, if it was tested above the threshold of detection at least once), while ultra sensitive RTQ-PCR was performed only once. As shown in table 2, 14 samples were detectable by both methods, while 4 and 5 samples were tested positive only by ultra sensitive RTQ-PCR and the dHBV test, respectively. These qualitative findings suggest that the in-house assay performed equally well as the commercial test, although the samples were tested multiple times by the latter method.

**Table 1:** Epidemiological, molecular and serological features of 27 samples obtained from 22 Blood Donors with Occult Hepatitis B Infection*

Patient ID	Sample	dHBV^1^	In-house Assay	IU/mL	aanti-HBc (IgM)	anti-Hbs	anti-HBe
Blood donor 1	1	+	+	69.2	-	-	-
	2	+	+	89.1	-	+	
Blood donor 2	3	+	-		-	-	-
Blood donor 3	4	+	-		-	-	-
Blood donor 4	5	+	+	10.2	-	+	-
	6	+	+	40.7	-	+	-
Blood donor 5	7	+	+	25.1			
	8	-	+	97.7	-	+	-
Blood donor 6	9	+	+	46.8	-	-	+
Blood donor 7	10	+	+	25.1	-	+	+
Blood donor 8	11	+	-		-	-	+
Blood donor 9	12	+	+	47.9	-	-	+
Blood donor 10	13	+	+	346.7	-	-	+
Blood donor 11	14	+	+	26.9	-	+	-
Blood donor 12	15	-	-		-	-	+
Blood donor 13	16	+	-		-	+	-
Blood donor 14	17	+	+	13.5	-	+	-
Blood donor 15	18	+	+	27.5	-	+	-
Blood donor 16	19	-	-		-	-	+
Blood donor 17	20	-	+	15.8	-	+	+
	21	-	-		-	+	+
Blood donor 18	22	+	+	41.7	-	-	+
	23	-	-		-	-	+
Blood donor 19	24	-	+	25.1	-	+	+
Blood donor 20	25	+	+	91.2	-	-	-
Blood donor 21	26	+	-		-	-	-
Blood donor 22	27	-	+	24.0	+	+	+

^1^dHBV: Procleix Ultrio discriminatory HBV test

*All subjects were HBsAg(-)/HBeAg(-)/IgG-anti-HBc(+)

**Table 2:** Efficiency of the ultra sensitive RTQ-PCR and HBV Procleix Ultrio discriminatory HBV tests (dHBV) on 27 samples collected from 22 individuals with occult Hepatitis B

Ultra sensitive RTQ-PCR	dHBV		
		
	+ (n. %)	- (n. %)	Total
+	14 (77.8%)	4 (22.2%)	18 (66.7%)
-	5 (55.6%)	4 (44.4%)	9 (33.3%)

**Total**	19 (70.4%)	8 (29.3%)	27 (100%)

### Calibration of the HBV-DNA values obtained with the ultra sensitive RTQ-PCR against the WHO 1st International Standard for HBV-DNA

**Figure 1:**
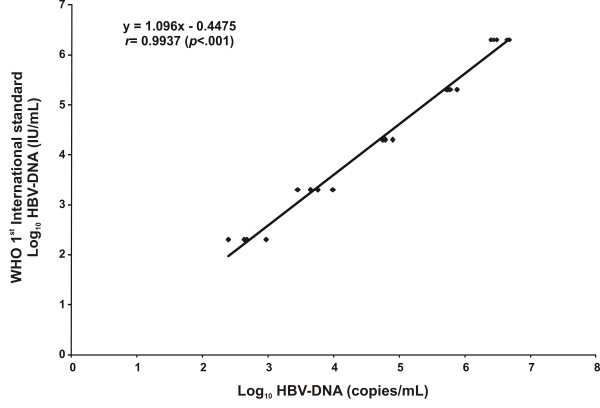
**Calibration of the HBV-DNA values using the ultra-sensitive RTQ-PCR assay versus the WHO International Units**. Correlation plot of the expected (log_10 _HBV-DNA IU/mL) versus the estimated values by ultra sensitive RTQ-PCR (log_10 _HBV-DNA copies/mL) tested on 5 serial concentrations of the WHO International standard ranging between 2 × 10^2 ^-2 × 10^6 ^IU/mL by using the plasmid DNA as a standard.

The calibration of the in-house assay was done against the WHO 1^st ^International Standard for HBV-DNA (OptiQuant^® ^HBV-DNA Quantification Panel, Accrometrix Europe B.V.) DNA was extracted and HBV-DNA was tested in 5 replicates by using the plasmid DNA as a standard. The correlation plot of the expected (log_10 _HBV-DNA IU/mL) versus the estimated values by ultra sensitive RTQ-PCR log_10 _HBV-DNA copies/mL) is shown in Fig. 1. The fitted regression lines between IU/mL and copies/mL was given by the following equation: log_10 _(IU/mL) = -0.4475 + 1.0096·log_10_(ultra sensitive RTQ-PCR copies/mL), suggesting that in our setting, 1 IU/mL = 2.8 copies/mL. The 95% CI for the estimated intercept was -0.69, -0.21. The 95% CI for the estimated slope was: 0.96, 1.06. The correlation coefficient between the expected and the estimated values was very good *r *= 0.9937 (*p *< .001) (Fig. 1).

### Comparison of the ultra sensitive RTQ-PCR with the previous HBV-DNA test

To assess the correlation between the previous and the new assay, we re-tested 25 previously quantified samples. The average log_10 _HBV-DNA was 5.38 copies/mL (range, 2.31 to 10.51 log_10 _copies/mL). Importantly, the linearity was very good between the HBV-DNA values quantified by the two assays (*r *= 0.985, *p *< .001) over the whole range of quantification. The fitted regression line between the ultra sensitive RTQ-PCR and the previous test was: log_10 _HBV-DNA (copies/mL) with the ultra sensitive RTQ-PCR = -0.531 + 1.015x log_10 _(copies/mL) with the previous real-time PCR test. The 95% CI for the estimated intercept was -0.92, -0.14. The 95% CI for the estimated slope was: 0.95, 1.08 (data not shown).

### Comparison of the ultra sensitive RTQ-PCR with TaqMan

Among the 94 samples tested with the TaqMan test, 85 (90.4%) were found to be above the threshold of detection, while a higher sensitivity was observed for the ultra sensitive RTQ-PCR method (89 samples tested positive out of 94, 94.7%). As shown in Table 3, 83 (88.3%) samples were detectable by both methods, while 6 (6.4%) and 2 (2.1%) samples were tested positive only by the ultra sensitive RTQ-PCR and the TaqMan test, respectively. These qualitative findings suggest that the new version of the in-house assay performed equally well as the commercially available TaqMan Test (McNemar's p = 0.157). The in-house assay found positive six samples (ranging from 1.37 to 1.83 log_10 _IU/mL) with undetectable viral load levels with COBAS TaqMan HBV Test, while it did not quantify two samples (with viral load levels 1.08 and 1.77 log_10 _IU/mL, respectively).

**Table 3:** Efficiency of the ultra sensitive RTQ-PCR and COBAS TaqMan Test on 94 randomly selected samples with Hepatitis B

Ultra sensitive RTQ-PCR	COBAS TaqMan Test		
		
	+ (n. %)	- (n. %)	Total
+	83 (88.3%)	6 (6.4%)	89 (94.7%)
-	2 (2.1%)	3 (3.2%)	5 (5.3%)

**Total**	85 (90.4%)	9 (9.6)	94 (100%)

**Figure 2:**
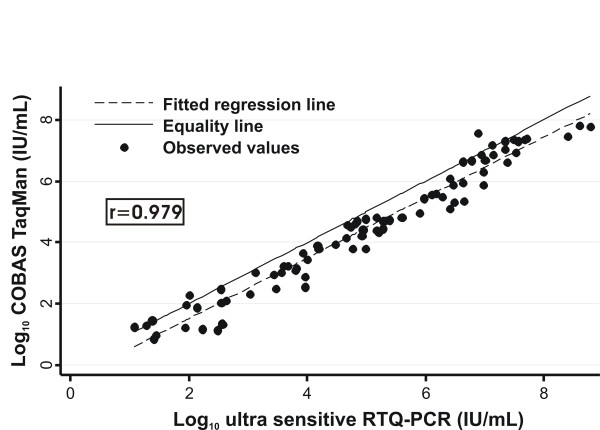
**Comparison of the HBV-DNA values estimated using COBAS TaqMan Test and ultra-sensitive RTQ-PCR**. Correlation plot of log_10 _HBV-DNA IU/mL as determined by ultra sensitive RTQ-PCR and COBAS TaqMan Test in 83 specimens detectable by both assays. Superimposed the regression fitted line (discontinuous line) and the line of equality (diagonal constant line).

Fig. 2 shows the scatter plot of log_10 _HBV-DNA IU/mL determined by the ultra sensitive RTQ-PCR and the TaqMan assays, using specimens with detectable HBV-DNA by both assays (*N *= 83). The results of the two assays were linearly correlated (*r *= 0.979, *p *< 0.001). The fitted regression line differs significantly from the line of equality and was described by the equation: log_10 _(TaqMan, IU/mL) = -0.457 + 0.987 log_10 _(ultra sensitive RTQ-PCR, IU/mL) (joint test of intercept = 0 and slope = 1: p < 0.001).

## Discussion

Sensitive detection and quantification of HBV-DNA is essential for monitoring response to therapy and, also, for assessment of occult HBV infection. Therefore, ultra-sensitive assays for HBV-DNA quantification are needed in clinical practice.

In the current study, we describe the characteristics of a new ultra sensitive in-house RTQ-PCR performed in a LightCycler^® ^2.0 instrument (Roche, Molecular Biochemicals, Mannheim, Germany). The new assay showed improved sensitivity of 22 and 8 IU/mL as 95% and 50% detection end-points, respectively, versus the 94 IU/mL (250 copies/mL) of the previously reported quantitative test [12]. The sensitivity of the assay was improved mainly because of a larger volume of HBV-DNA used in ultra sensitive RTQ-PCR, extracted also from a larger volume of serum/plasma. Additionally, the new assay was carried out in the LightCycler^® ^2.0 that shows improved performance than the LightCycler^® ^1.0. We should note that the sensitivity of the assay can be potentially improved by using a larger than 0.5 mL volume of plasma, after a concentration step performed before the standard procedure. Moreover, the plasmid standard calibrated against the WHO 1^st ^International standard, thus suggesting that the HBV-DNA values are reported in the international format (IU/mL).

Importantly, the ultra sensitive RTQ-PCR performed equally well as the qualitative dHBV assay tested on low-titer HBV-DNA samples collected from individuals with occult Hepatitis B. These findings are in accordance with the previously reported 95% detection limit of the dHBV, 19 IU/mL [16], which is almost identical to the 95% detection limit (22 IU/mL) of the ultra sensitive RTQ-PCR assay. These findings suggest that the ultra sensitive RTQ-PCR assay is equally reliable as the dHBV for detecting HBV-DNA in low-titer samples as those collected from patients with occult Hepatitis B. We should note however that the ultra sensitive RTQ-PCR is a quantitative test and therefore it shouldn't be used for diagnostic purposes.

The HBV-DNA quantified values between the ultra sensitive RTQ-PCR and the previously reported HBV-DNA test correlated very well, suggesting that the new version of the test can be utilized in occasions in which the original test was used. Furthermore, the ultra sensitive RTQ-PCR was found to be highly correlated with commercial available COBAS TaqMan HBV Test (*r *= 0.979, *p *< 0.001).

## Conclusion

In the era of highly potent antivirals (e.g. tenofovir, entecavir) approved for the treatment of chronic Hepatitis B, it is of crucial importance to utilize highly sensitive assays for detecting and quantifying HBV-DNA. We report the new ultra sensitive assay, using molecular beacon as a detection system, with remarkable analytical and clinical sensitivity, calibrated against the WHO 1^st ^International standard.

## Competing interests

The authors declare that they have no competing interests.

## Authors' contributions

DP participated to the study design and coordination, he prepared the manuscript and the reply to the reviewer's comments; AB and CH carried out the experiments; AK, ZM, HH, AV were responsible for the part of the study concerning the occult Hepatitis B infection; VS did the statistical analysis; AH was the study coordinator and participated to the writing and editing of the manuscript. All authors read and approved the final manuscript.

## References

[B1] Berger A Preiser W Doerr HW The role of viral load determination for the management of human immunodeficiency virus. hepatitis B virus and hepatitis C virus infection J Clin Virol 2001 20 23 30 10.1016/S1386-6532(00)00151-7 11163579

[B2] Locarnini S Birch C Antiviral chemotherapy for chronic hepatitis B infection: lessons learned from treating HIV-infected patients J Hepatol 1999 30 536 550 10.1016/S0168-8278(99)80118-4 10190742

[B3] Perrillo RP Schiff ER Davis GL Bodenheimer HC Jr Lindsay K Payne J A randomized. controlled trial of interferon alfa-2b alone and after prednisone withdrawal for the treatment of chronic hepatitis B. The Hepatitis Interventional Therapy Group N Engl J Med 1990 323 295 301 2195346 10.1056/NEJM199008023230503

[B4] Zarski JP Kuhns M Berck L Degos F Schalm SW Tiollais P Brechot C Comparison of a quantitative standardized HBV-DNA assay and a classical spot hybridization test in chronic active hepatitis B patients undergoing antiviral therapy Res Virol 1989 140 283 291 10.1016/S0923-2516(89)80108-6 2772413

[B5] Zoulim F Mimms L Floreani M Pichoud C Chemin I Kay A New assays for quantitative determination of viral markers in management of chronic hepatitis B virus infection J Clin Microbiol 1992 30 1111 1119 1583107 10.1128/jcm.30.5.1111-1119.1992 PMC265234

[B6] Abe A Inoue K Tanaka T Kato J Kajiyama N Kawaguchi R Quantitation of hepatitis B virus genomic DNA by real-time detection PCR J Clin Microbiol 1999 37 2899 2903 10449472 10.1128/jcm.37.9.2899-2903.1999 PMC85408

[B7] Jardi R Rodriguez F Buti M Costa X Cotrina M Valdes A Quantitative detection of hepatitis B virus DNA in serum by a new rapid real-time fluorescence PCR assay J Viral Hepat 2001 8 465 471 10.1046/j.1365-2893.2001.00322.x 11703579

[B8] Kapke GE Watson G Sheffler S Hunt D Frederick C Comparison of the Chiron Quantiplex branched DNA (bDNA) assay and the Abbott Genostics solution hybridization assay for quantification of hepatitis B viral DNA J Viral Hepat 1997 4 67 75 10.1046/j.1365-2893.1997.00127.x 9031068

[B9] Krajden M Minor J Cork L Comanor L Multi-measurement method comparison of three commercial hepatitis B virus DNA quantification assays J Viral Hepat 1998 5 415 422 10.1046/j.1365-2893.1998.00129.x 9857351

[B10] Lai VC Guan R Wood ML Lo SK Yuen MF Lai CL Nucleic acid-based cross-linking assay for detection and quantification of hepatitis B virus DNA J Clin Microbiol 1999 37 161 164 9854083 10.1128/jcm.37.1.161-164.1999 PMC84196

[B11] Loeb KR Jerome KR Goddard J Huang M Cent A Corey L High-throughput quantitative analysis of hepatitis B virus DNA in serum using the TaqMan fluorogenic detection system Hepatology 2000 32 626 629 10.1053/jhep.2000.9878 10960459

[B12] Paraskevis D Haida C Tassopoulos N Raptopoulou M Tsantoulas D Papachristou H Development and assessment of a novel real-time PCR assay for quantitation of HBV DNA J Virol Methods 2002 103 201 212 10.1016/S0166-0934(02)00033-2 12008014

[B13] Pas SD Fries E De Man RA Osterhaus AD Niesters HG Development of a quantitative real-time detection assay for hepatitis B virus DNA and comparison with two commercial assays J Clin Microbiol 2000 38 2897 2901 10921947 10.1128/jcm.38.8.2897-2901.2000 PMC87141

[B14] Lole KS Arankalle VA Quantitation of hepatitis B virus DNA by real-time PCR using internal amplification control and dual TaqMan MGB probes J Virol Methods 2006 135 83 90 10.1016/j.jviromet.2006.02.004 16551481

[B15] Katsoulidou A Paraskevis D Magiorkinis E Moschidis Z Haida C Hatzitheodorou E Molecular characterization of occult hepatitis B cases in Greek blood donors J Med Virol 2009 81 815 825 10.1002/jmv.21499 19319945

[B16] Katsoulidou A Moschidis Z Sypsa V Chini M Papatheodoridis GV Tassopoulos NC Analytical and clinical sensitivity of the Procleix Ultrio HIV-1/HCV/HBV assay in samples with a low viral load Vox Sang 2007 92 8 14 10.1111/j.1423-0410.2006.00857.x 17181585

